# Supplementary material for: Reduced contribution of sulfur to the mass extinction associated with the Chicxulub impact event
Source: Nat Commun. 2025 Jan 16;16:620. doi: 10.1038/s41467-024-55145-6 (PMC11739411; doi:10.1038/s41467-024-55145-6)
Supplement: Supplementary file 1 — Supplementary Information [file 41467_2024_55145_MOESM1_ESM.pdf]

Supplementary Information for

Reduced contribution of sulfur to the mass extinction associated with the Chicxulub impact event

Authors: Katerina Rodiouchkina, Steven Goderis, Cem Berk Senel, Pim Kaskes, Özgür Karatekin, Michael Ernst Böttcher, Ilia Rodushkin, Johan Vellekoop, Philippe Claeys, Frank Vanhaecke.

Sulfur concentration and isotopic composition in the target

The highest bulk S concentrations and $\delta^{34}S$ values in the drill cores are observed for the Yax-1 and UNAM-6 cores (20-23 wt% and $\delta^{34}S$ = 18.5 to 19.5 ‰), followed by the UNAM-5 and 7 cores (6-10 wt% and $\delta^{34}S$ = 18.0 to 18.8 ‰), then the Y6 core (0.5-2 wt% and $\delta^{34}S$ = 17.1 to 17.9 ‰), while the lowest are observed for the M0077A core (0.002-4 wt% and $\delta^{34}S$ = -17.2 to 8.5 ‰) (Fig. 1B; Table S1). Pure anhydrite and gypsum have S concentrations of 19 to 24 wt% and their $\delta^{34}S$ values mirror the $\delta^{34}S$ value of the sulfate in the water source from which it originates[1]. All the onshore drill cores located in the southern semicircle composing the Chicxulub impact structure (Yax-1, Y6, UNAM-5-7) have similar $\delta^{34}S$ values that agree well with the previously determined average $\delta^{34}S$ value of 18.3‰ for the Yax-1 and Y6 drill cores (ranging between 18.0 to 19.8‰)[2] and the seawater sulfate $\delta^{34}S$ values ranging between 17 to 19‰ at the end of the Cretaceous[1,3]. The high S concentrations observed for the Yax-1 and UNAM-6 indicate pure anhydrite, while the other represent a mixed composition with high inclusion of evaporites as even the lower S concentrations found in the suevite and impact melt rock sections of the Y6 core show similar $\delta^{34}S$ values (Fig. S2).

The latest offshore M0077A drill core is clearly an outlier when comparing lithology as well as bulk S concentrations and $\delta^{34}S$ values around the K-Pg boundary to those of the previously characterized Yax-1, Y6, and UNAM 5-7 drill cores. In a previous study[4], bulk powder X-ray diffraction (XRD) analysis of the M0077A drill core recorded total percentages of gypsum and anhydrite of 0.73 and 0.04%, respectively. Petrographic examination showed no anhydrite or gypsum minerals present in the M0077A drill core stratigraphy[4]. In another study[5], the bulk S concentrations determined using micro

X-ray fluorescence spectrometry (µXRF) were <7,000 µg g^{-1} throughout the M0077A drill core, except for outliers up to 80,000 µg g^{-1} found in the transitional unit and upper impact melt rock section, which are attributed to the occurrence of pyrite and other sulfides[5]. This is largely consistent with what was observed in the present study, where the highest bulk S concentrations are identified in the bedded suevite and the post-impact units around the K-Pg boundary, but remain <40,000 µg g^{-1}. The bulk $\delta^{34}S$ values determined for these units range from -9 to -5 ‰, and are clearly distinct from the $\delta^{34}S$ values determined for the anhydrite sections in the other onshore drill cores from the southern part of the Chicxulub impact structure (Fig. S2).

The highest bulk $\delta^{34}S$ value in the M0077A drill core profile is observed in the middle part of the graded suevite unit, with an average value of 8.5 ‰ (Fig. 1B; Table S1), indicating the presence of evaporites. Further, this section also shows the largest shift (14.0‰) between the bulk S isotope ratio and the sulfide-specific isotope ratio ($\delta^{34}S_{sulfide}$=-5.5‰) and has a calculated sulfate-specific isotope ratio ($\delta^{34}S_{sulfate}$=14.7‰, assuming that all S in this section is either related to sulfides or sulfates, Table S3) close to the bulk S isotope ratio range found in the evaporite-containing sections located in the five onshore drill cores ($\delta^{34}S$=17.1-19.5‰, Yax-1, Y6, and UNAM-5-7). However, this unit does not coincide with a high S concentration (500 µg g^{-1}), which would be expected if a significant amount of evaporite was mixed in. This S concentration level of the graded suevite unit is approximately 80-fold lower than that of the bedded suevite unit (39,000 µg g^{-1}) and between 10- to 500-fold lower than the S concentrations of the five onshore drill cores (5000-230,000 µg g^{-1}) (Fig.1B; Table S1).

The TRIS fraction throughout the M0077A drill core (22-86%) is higher than the TRIS fraction for the Y6 drill core (≤1 %) (Fig. S5 and Table S3) that shows the lowest bulk S concentration of the onshore drill cores. Still the lower TRIS fraction (29%) coinciding with a higher $\delta^{34}S$ value for the graded suevite unit (Fig. 1B and S5; Table S1 and S3) could be linked to anhydrite and gypsum present in this section. In general, the units with lower TRIS fractions (<36%) display higher bulk $\delta^{34}S$ values,

with values of 0.7-2.2‰ observed for the post-impact and lower LIMB units, while the values with high TRIS fractions (>50%) show a large spread in $\delta^{34}S$ values between -17.2 and 0.3 ‰ (Fig. 1B and S5; Table S1 and S3).

The lowest bulk $\delta^{34}S$ values are found in the Paleogene sediment (-9.1 to -5.0 ‰), bedded suevite (-7.4 to -5.3 ‰), UIM (-15.8 to -0.27 ‰), dolerite (-11.6 to -1.06 ‰), and upper LIMB (-17.2 ‰) units. This is in agreement with the previously published pyrite $\delta^{34}S$ value for the Paleogene sediment (608.48-618.98 mbsf, -32.9 to 0.9 ‰)[6] and for the suevite (618.72-685.47 mbsf, -35.9 to -6.2 ‰)[7] units of the M0077A drill core (Fig. 1B; Table S1). These low $\delta^{34}S$ values observed in the M0077A drill core are suggested by Kring *et al.* 2020 [8], 2021 [7] to result from late-stage microbial reduction of S in the impact-generated hydrothermal systems, both in the porous, permeable subsurface rock of the crater and in the water column above. Similarly, a large negative S isotope fractionation between sulfate and sulfide, and gypsum and marcasite between 44 and 78‰ was observed at Haughton Crater in the Canadian High Arctic, and a sulfur isotope ratio spread of 77‰ was reported for the Miocene German Nördlinger Ries crater[9], suggested to result from thermochemical sulfate reduction, microbial sulfate reduction, hydrothermal equilibrium fractionation, or any combination thereof. In the deepest LIMB (1242-1334 mbsf, 0.32 to 3.0 ‰) unit, the bulk $\delta^{34}S$ values compare well with previously published pyrite $\delta^{34}S$ values for samples at a similar depth in the M0077A drill core (1313.92 mbsf, -3.1 to -1.8 ‰)[7] (Fig. 1B; Table S1). These near-zero $\delta^{34}S$ values found deep within the drill core are similar to the $\delta^{34}S$ value for the upper mantle of -1.40 ± 1.00‰ (2SD) based on mid-ocean-ridge basalts[10–13], indicating that the majority of the S in this unit is likely related to igneous rocks.

In line with previous studies, the S concentrations and $\delta^{34}S$ values determined for the new M0077A drill core also show that it is largely devoid of evaporites in contrast to previous onshore drill cores[4,5]. The reason for this lack of evaporite in the M0077A core is not yet fully understood. It has been suggested that this absence of evaporites can be explained by the non-porous evaporites present

in the target rock not vaporizing efficiently and more likely to fragment into larger clasts, transported as low-velocity ejecta and deposited outside of the peak ring[4]. A heterogeneous distribution of evaporites within the sedimentary layers of the pre-impact target rock[14] can also be advocated; considerable variation in the evaporite proportion could be expected considering the size of the structure. Therefore, it might be interesting to focus future drilling projects on the more northern part of the crater in order to test this hypothesis and better assess the exact amount of evaporite involved in the event.

Traces of possible evaporite content are present in the offshore M0077A drill core as higher $\delta^{34}S$ values and lower TRIS fractions are observed in the graded suevite (Fig. 1B and S5; Table S1 and S3). As the S concentrations are low, the measured signals likely do not exclusively reflect the target bedrock, but are potentially influenced by later post-impact processes, such as microbial S reduction generated by the hydrothermal system active within the Chicxulub peak ring[7]. Therefore, the five onshore drill cores are preferably used to determine the S isotopic 'fingerprint' in the target rock as these have higher S concentrations. Moreover, similar $\delta^{34}S$ values are observed for the unshocked anhydrite (Yax-1, UNAM-5, and UNAM-7) and deposited anhydrite clasts in the K-Pg boundary sediments (UNAM-6) (Table S1). Here, the bulk $\delta^{34}S$ value of the target rock is determined based on the $\delta^{34}S$ values observed for the evaporite-containing lithological units in five different cores drilled in and near the Chicxulub impact structure (Yax-1, Y6, and UNAM-5-7), which yield a mean value of 18.5 ± 1.4‰ (2SD).

K-Pg boundary ejecta deposition sites: Brazos River

The S profiles obtained for the previously studied[15] marine Brazos River site display a positive peak in S concentration coinciding with a positive $\delta^{34}S$ shift (6,000-30,000 µg g^{-1} and -40 ‰ to -33 ‰), possibly also indicating an influx of impact-deposited target anhydrite. The TRIS content in selected samples

from the Brazos River K-Pg site profile ranges from 2,600 to 12,000 μg g^{-1}, corresponding to 34-100% of the bulk S concentration (Table S3), with the lowest TRIS fraction coinciding with the most ^{34}S-enriched sample at the K-Pg boundary (Table S2), further indicating that the observed increase in $\delta^{34}S$ is related to evaporate-originating sulfate deposition. However, in contrast to the other sites, the Brazos River S-profiles are jagged (Fig. 2D), indicating heterogenous S input in these K-Pg sediments. This is further demonstrated by large variations in the bulk S concentration and $\delta^{34}S$ value for multiple sub-samples taken from the same location depth (Fig. 2D). A similar jagged behavior is also observed in siderophile element concentrations (Fig. S8) and Ir concentration profiles, at sites across the Gulf of Mexico, indicative of wash-in from impact tsunamis[16,17]. Consequently, this observed heterogeneity likely results from the mixing of S deposition from the atmosphere and the wash-in of low-velocity clasts from within the crater due to the proximity to the impact site. If this site is used for the calculation of impact-released S into the atmosphere, this dual source of S causes an overestimation, which excludes this site from being representative for these types of calculations. This likely accounts for the large uncertainty accompanying the estimate obtained in this study for the Brazos River site (337 ± 285 Gt S).

Mass-independent fractionation

Complementary to S concentrations and $\delta^{34}S$ values, impact-vaporized S depositions can be further traced using mass-independent fractionation (MIF) tracers ($\Delta^{33}S$ and $\Delta^{36}S$), as demonstrated by Junium *et al.* 2022 [15]. A number of gas phase reactions can lead to MIF of S isotopes and these can be traced using the $\Delta^{33}S$ and $\Delta^{36}S$ values. Contributions to MIF include, but are not limited to, stabilization of asymmetric isotopologue intermediate species during extensive biomass burning[18] and/or the self-shielding effect of $^{32}SO_2$ compared to other isotopologues during photolysis with UV light due to its higher relative abundance in the gas column[19]. Generally, these MIF signatures are small and poorly preserved in sediments due to redox cycling of S deposited from the atmosphere in an oxidizing

Paleogene atmosphere[20]. Preservation of atmospheric MIF signals in sediments therefore requires a sufficiently large quantity of deposition in a short amount of time to transcend the S isotope ratios of the local sulfate flux to the sediments[15], in line with the hypothesis of massive amounts of impact-vaporized S into the atmosphere.

Similarly to Junium *et al.* 2022 [15], negative mass-independent $\Delta^{33}S$ anomalies were observed for the K-Pg boundary section of the Brazos River site (Fig. S4), indicating that a portion of the deposited S had resided in the atmosphere. These negative mass-independent anomalies are also observed to coincide with the positive S concentration peak for the Stevns Klint site, indicating that impact-deposition of S might have occurred at this site, but that its S isotope ratio signature has been overprinted by post-impact processes. For the Caravaca site, this mass anomaly is less pronounced, probably due to the poor peak resolution owing to lower post-impact sedimentation rates (0.2–0.5 cm/k.y) compared to the other two marine sites (1–3 cm/k.y)[21], but the $\Delta^{33}S$ values at the K-Pg boundary are slightly lower than those measured in the Paleocene sediments. However, many of these observations are provisional as they are based on $\Delta^{33}S$ values that are = 0 ‰ within the expanded uncertainty of the method. The only samples for which the $\Delta^{33}S$ values are ≠ 0 % taken into account the expanded uncertainty coincide with the S offset peaks in the Brazos River (-0.17 ± 0.14 ‰, -0.20 ± 0.15 ‰, -0.27 ± 0.11 ‰, -0.17 ± 0.09 ‰) and Stevns Klint (-0.13 ± 0.12 ‰, -0.15 ± 0.10 ‰) K-Pg boundary profiles, as well as for the Elles (-0.25 ± 0.23 ‰) K-Pg boundary clay sample (Table S2), supporting the hypothesis that a portion of the S in these K-Pg sediments originated from atmospheric S. However, this does not rule out influence of other post-impact processes in the K-Pg sediment of these sites.

Table S1. Bulk S concentration and isotopic composition in different drill cores of the Chicxulub impact structure. Expanded uncertainties (U) are presented for the bulk isotopic compositions and n represents the number of separate sample preparations.

Location	Core	Sample ID	Depth mbsf	Lithological unit	Bulk S μg g^{-1}	SD μg g^{-1}	$\delta^{34}S$	U	$\delta^{33}S$	U	$\Delta^{33}S$	U	n
Mexico	IODP-ICDP Expedition 364	39_3_11_12	615.88	Paleogene sediments	550	20	6.08	0.23	3.10	0.40	-0.03	0.39	1
		40_1_30_36	616.53	Paleogene sediments	12000	500	-9.14	0.36					1
		40_1_38_44	616.63	Paleogene sediments	10500	500	-5.02	0.26					1
		40_1_38_44	616.67	Transitional unit	1200	100	2.85	5.22	2.80	0.39	-0.01	0.38	2
		40_1_49_50	616.73	Transitional unit	1430	60	2.17	0.21	1.14	0.39	0.03	0.37	1
		40_1_80_82	617.04	Transitional unit	1130	40	3.02	0.24	1.59	0.40	0.04	0.37	1
		40_1_106_110	617.33	Transitional unit	11900	100	-5.88	0.28	-2.84	0.60	0.18	0.55	1
		40_1_111_113	617.35	Bedded suevite unit	39000	1800	-5.30	0.30	-2.69	0.43	0.03	0.39	1
		40_2_100_103	618.67	Bedded suevite unit	8240	80	-7.35	0.24	-3.69	0.39	0.10	0.37	1
		55_3_8_14	664.49	Graded suevite unit	460	20	8.50	0.22	4.32	0.39	-0.06	0.37	1
		68_1_67_70	692.13	Graded suevite unit	490	20	6.32	0.22	3.24	0.40	-0.01	0.38	1
		81_3_30_32	710.29	Non-graded suevite unit	610	30	-0.32	0.25	-0.21	0.38	-0.04	0.37	1
		90_2_48_50b	730.29	Granitoid clast inside UIM (Unit 3A)	1320	60	-15.77	0.22	-8.16	0.39	-0.05	0.38	1
		91_3_18_20	734.25	Upper impact melt rock unit (UIM, Unit 3A)	700	30	-0.27	0.24	-0.14	0.39	-0.01	0.37	1
		139_1_6_8	853	Dolerite	1830	80	-11.60	0.18	-5.96	0.23	0.01	0.28	1
		162_2_99.5_101.5	914.63	Dolerite	1660	70	-1.06	0.17	-0.68	0.31	-0.12	0.33	1
		164_3_14_16.5	920.36	Dacite	91	2	-0.50	0.35	-0.30	0.36	-0.07	0.28	2
		192_1_56_58	997.65	Lower impact melt-bearing unit (LIMB)	430	20	-17.21	0.21	-8.79	0.39	0.07	0.37	1
		264_1_53_55	1212.67	Granitoid	16	6	6.12	0.22	3.01	0.39	-0.14	0.38	1
		273_2_78_80	1242	LIMB	620	30	1.47	0.22	0.74	0.39	-0.01	0.38	1
		276_3_93_95	1252.33	Metamorphic clast (gneiss/amphibolite)	810	30	2.10	0.28	1.13	0.48	0.05	0.45	1
		282_1_80_82	1268.74	LIMB	120	10	2.98	0.30	1.72	0.43	0.20	0.45	2
		283_2_46_48	1272.97	LIMB	490	20	0.32	0.16	0.12	0.32	-0.05	0.36	1
		303_3_17.5_19.5	1334.33	LIMB	1450	60	0.66	0.21	0.41	0.52	0.07	0.51	1
Mexico	Yucatán-6	Y6_N14_P15	1208-1211	Suevite	5000	200	17.06	0.38	8.59	0.40	-0.11	0.23	3
	Yucatán-6	Y6_N17	1295.95-1299	Impact melt rock	22600	300	17.93	0.31	9.14	0.26	-0.10	0.20	3
Mexico	UNAM 5	500.55-500.65	500.55	Evaporite (anhydrite)	57700	2500	17.96	0.38					3
Mexico	UNAM 6	107_385.85-385.95	385.85	Evaporite clast	199200	8800	19.48	0.32					4
Mexico	UNAM-7	135_381.40-381.50	381.4	Evaporite (anhydrite)	102700	4500	18.80	0.31					4
	UNAM-7	97_267.40-267.50	267.4	Evaporite (anhydrite)	81700	3600	18.58	0.40					4
Mexico	Yaxcopoil-1	1576a	1080.35	Cretaceous megablock	229000	16000	19.01	0.30	9.78	0.24	-0.05	0.18	9
	Yaxcopoil-1	1568a	1057.99	Cretaceous megablock	229000	13000	18.47	0.22	9.43	0.27	-0.10	0.23	6
	Yaxcopoil-1	1606a	1165.24	Cretaceous megablock	200500	8900	19.24	0.47	9.75	0.24	-0.02	0.23	8

Table S2. Bulk S concentration and isotopic composition in different K-Pg boundary deposition sites. Expanded uncertainties (U) are presented for the bulk isotopic compositions and n represents the number of separate sample preparations.

Location	Section/Core	Sample ID	Depth cm	Lithological unit	Bulk S $\mu g\ g^{-1}$	SD $\mu g\ g^{-1}$	$\delta^{34}S$	U	$\delta^{33}S$	U	$\Delta^{33}S$	U	n
Denmark	Stevns Klint A	SK KSA 50	27.5	K-Pg section	360	30	20.88	0.18	10.74	0.09	-0.01	0.09	1
	Stevns Klint A	SK KSA 40	19.5	K-Pg section	410	40	17.40	0.18	8.92	0.20	-0.04	0.17	1
	Stevns Klint A	SK KSA 43	16.5	K-Pg section	310	30	15.98	0.18	8.21	0.20	-0.02	0.22	1
	Stevns Klint A	SK KSA 45	14.5	K-Pg section	770	70	-19.73	0.17	-10.14	0.24	0.02	0.23	1
	Stevns Klint A	SK KSA 47	12.5	K-Pg section	8700	1400	-39.38	0.37	-20.41	0.12	-0.13	0.12	2
	Stevns Klint A	SK KSA 20	9.5	K-Pg section	9000	100	-37.54	0.16	-19.50	0.16	-0.16	0.17	1
	Stevns Klint A	SK KSA 23	7.5	K-Pg section	10500	1800	-36.37	0.37	-18.89	0.08	-0.15	0.10	2
	Stevns Klint A	SK KSA 2	0.75	K-Pg section	360	30	-8.52	0.21	-4.27	0.23	0.12	0.19	1
	Stevns Klint A	SK KSA 1b	0.75	K-Pg section	590	50	-7.20	0.16	-3.56	0.30	0.15	0.30	1
	Stevns Klint A	SK KSA 1	0.015	K-Pg section	4300	410	-12.23	0.18	-6.25	0.17	0.05	0.16	1
	Stevns Klint A	SK KSB 98	-4.5	K-Pg section	220	10	18.81	0.18	9.61	0.10	-0.08	0.12	2
Spain	Caravaca	SM75 510	22	K-Pg section	210	10	19.07	0.14	9.80	0.20	0.03	0.19	1
	Caravaca	SM75 507	7	K-Pg section	410	20	-13.77	0.15	-7.09	0.17	0.00	0.14	1
	Caravaca	504	1	K-Pg section	550	20	-17.85	0.12	-9.17	0.12	0.02	0.11	1
	Caravaca	503C	0.25	K-Pg section	640	30	-13.63	0.14	-6.97	0.17	0.05	0.14	1
	Caravaca	503B	0	K-Pg section	780	30	-32.29	0.19	-16.63	0.15	0.00	0.11	1
	Caravaca	501A	-1	K-Pg section	260	10	18.73	0.12	9.60	0.14	-0.04	0.16	2
	Caravaca	SM75 515	-24	K-Pg section	370	20	-8.99	0.17	-4.74	0.18	-0.11	0.16	2
	Caravaca	SM75 517	-44	K-Pg section	580	10	-18.68	0.17	-9.69	0.24	-0.07	0.20	1
Texas, USA	Brazos River	JV-BR 18	100	K-Pg section	4600	100	-39.56	0.38					1
	Brazos River	JV-BR 41	75	K-Pg section	2900	100	-39.87	0.21	-20.63	0.14	-0.10	0.17	1
	Brazos River	JV-BR 16	65	K-Pg section	5100	100	-40.26	0.43					1
	Brazos River	JV-BR 14	50	K-Pg section	6700	200	-40.32	0.43					1
	Brazos River	JV-BR 28	45	K-Pg section	10100	200	-40.79	0.20	-21.19	0.25	-0.20	0.26	1
	Brazos River	JV-BR 9	40	K-Pg section	9000	200	-40.35	0.36					1
	Brazos River	JV-BR 38	28	K-Pg section	5800	100	-37.17	0.28					1
	Brazos River	JV-BR 49	18	K-Pg section	4400	100	-37.16	0.20					1
	Brazos River	JV-BR 19	15	K-Pg section	8800	200	-36.52	0.23	-18.98	0.17	-0.17	0.14	1
	Brazos River	JV-BR 68	13.4	K-Pg section	8600	50	-37.34	0.20	-19.44	0.15	-0.20	0.15	1
	Brazos River	JV-BR 60	10.9	K-Pg section	4300	100	-35.72	0.22					1
	Brazos River	JV-BR 30	5.5	K-Pg section	6000	100	-37.61	0.24	-19.64	0.12	-0.27	0.11	1
	Brazos River	JV-BR 50	2.5	K-Pg section	1610	40	-32.60	0.29					2
	Brazos River	JV-BR 51	1.5	K-Pg section	1560	40	-32.01	0.31					2
	Brazos River	JV-BR 66	0	K-Pg section	3580	80	-34.27	0.21					1
	Brazos River	JV-BR 36	-15	K-Pg section	27500	2200	-32.96	5.75	-19.51	0.25	-0.23	0.24	4
	Brazos River	JV-BR 29	-20	K-Pg section	3460	80	-35.20	0.40					2
	Brazos River	JV-BR 35	-26	K-Pg section	12900	300	-38.83	0.21	-20.09	0.09	-0.09	0.10	1
	Brazos River	JV-BR 22	-32	K-Pg section	8900	200	-38.47	0.49					1
	Brazos River	JV-BR 33	-40	K-Pg section	9800	200	-36.79	0.27	-19.06	0.14	-0.12	0.16	1
	Brazos River	JV-BR 20	-52.5	K-Pg section	14800	300	-39.18	0.24	-20.26	0.14	-0.08	0.11	1
	Brazos River	JV-BR 6	-125	K-Pg section	11000	200	-39.04	0.21	-20.27	0.10	-0.17	0.09	1
	Brazos River	JV-BR 7	-225	K-Pg section	12300	200	-36.68	0.16	-18.95	0.15	-0.08	0.15	1
N-Dakota, USA	Tanis	X-2761_1_0-1cm	12.5	Paleocene siltstone	1200	100	-0.80	0.18					1
	Tanis	X-2761_2_1-2cm	11.5	Paleocene siltstone	1800	100	-0.26	0.18					1
	Tanis	X-2761_3_2-3cm	10.5	Paleocene siltstone	2010	20	1.65	0.18					1
	Tanis	X-2761_4_3-4cm	9.5	Paleocene coal	7800	1100	4.36	0.20					2
	Tanis	X-2761_5_4-5cm	8.5	Paleocene coal	6500	1100	4.43	0.54					2
	Tanis	X-2761_6_5-6cm	7.5	Paleocene coal	7400	800	4.68	0.24					1
	Tanis	X-2761_7_6-7.25cm	6.375	Paleocene coal	6170	10	0.53	0.20					1
	Tanis	X-2761_8A_7.25-8cm	5.375	K-Pg tonstein	1720	10	-2.33	0.18					1
	Tanis	X-2761_8B_8-8.5cm	4.75	K-Pg tonstein	2570	10	-1.99	0.18					1
	Tanis	X-2761_8C_8.5-9cm	4.25	K-Pg tonstein	3300	100	-1.90	0.18					1
	Tanis	X-2761_9_9-10cm	3.5	Event deposit	600	10	-5.12	0.19					1
	Tanis	X-2761_10_10-11cm	2.5	Event deposit	640	20	-5.78	0.20					1
Haiti	Beloc	Goderis et al. (2013)[22]		K-Pg boundary clay	140	10	17.36	0.27	8.87	0.27	-0.07	0.24	1
Texas, USA	Brazos River	Goderis et al. (2013)[22]		K-Pg boundary clay	12000	100	-31.81	0.30	-16.52	0.20	-0.14	0.21	1
Colorado, USA	Long Canyon, Raton Basin	Goderis et al. (2013)[22]		K-Pg boundary clay	1460	10	4.01	0.29	2.06	0.17	-0.01	0.18	1
Wyoming, USA	Dogie Creek, Powder River Basin	Goderis et al. (2013)[22]		K-Pg boundary clay	3250	40	-0.89	0.30	-0.52	0.29	-0.06	0.23	2
Montana, USA	Brownie Butte, Hell Creek area	Goderis et al. (2013)[22]		K-Pg boundary clay	4170	40	-3.20	0.28	-1.71	0.18	-0.06	0.15	1
Montana, USA	Seven Blackfoot Creek, Hell Creek area	Goderis et al. (2013)[22]		K-Pg boundary clay	510	20	-1.98	0.22	-0.98	0.31	0.04	0.30	2
Italy	Frontale, Umbria-Marche	Goderis et al. (2013)[22]		K-Pg boundary clay	90	20	16.23	0.31	8.35	0.27	0.00	0.18	1
Italy	Fonte D'Olio, Umbria-Marche	Goderis et al. (2013)[22]		K-Pg boundary clay	170	20	15.81	0.26	8.22	0.37	0.08	0.38	1
Tunisia	Siliana	Goderis et al. (2013)[22]		K-Pg boundary clay	3580	40	12.72	0.30	6.29	0.26	-0.14	0.25	1
Tunisia	Elles	Goderis et al. (2013)[22]		K-Pg boundary clay	63200	600	18.00	0.26	9.02	0.26	-0.25	0.23	1

Table S3. Total reduced inorganic S (TRIS), TRIS-fraction (TRIS/bulk S concentration*100), sulfide-specific isotope ratio ($\delta^{34}S_{sulfide}$), isotope ratio difference between bulk and sulfide-specific sulfur isotope ratio ($\delta^{34}S_{bulk}$-$\delta^{34}S_{sulfide}$), and calculated sulfate-specific isotope ratio ($\delta^{34}S_{sulfate}$)[#] in different K-Pg boundary drill cores of the Chicxulub impact-structure and the Brazos River K-Pg boundary section.

Location	Section/Core	Sample ID	Depth cm	Lithological unit	TRIS µg g^{-1}	TRIS-fraction %	$\delta^{34}S_{sulfide}$ ‰	($\delta^{34}S_{bulk}$-$\delta^{34}S_{sulfide}$) ‰	$\delta^{34}S_{sulfate}$[#] ‰
Texas, USA	Brazos River	JV-BR 28	45	K-Pg section	4030	40	-42.00	1.2	-40.0
	Brazos River	JV-BR 19	15	K-Pg section	5060	57	-38.60	2.1	-33.7
	Brazos River	JV-BR 68	13.4	K-Pg section	8760	102	-38.80	1.5	-
	Brazos River	JV-BR 30	5.5	K-Pg section	2630	44	-35.60	-2.0	-39.2
	Brazos River	JV-BR 36	-15	K-Pg section	9430	34	-33.90	3.6	-28.3
	Brazos River	JV-BR 35	-26	K-Pg section	5680	44	-39.76	0.9	-38.1
	Brazos River	JV-BR 33	-40	K-Pg section	5180	53	-37.90	1.1	-35.5
	Brazos River	JV-BR 20	-52.5	K-Pg section	12450	84	-39.73	0.5	-36.4
	Brazos River	JV-BR 6	-125	K-Pg section	5380	49	-40.78	1.7	-37.4
	Brazos River	JV-BR 7	-225	K-Pg section	5270	43	-37.67	1.0	-35.9
			**mbsf**						
Mexico	IODP-ICDP Expedition 364	40_1_49_50	616.73	Transitional unit	510	36	-7.80	9.9	7.7
		40_2_100_103	618.67	Bedded suevite unit	6980	85	-9.20	1.9	2.9
		55_3_8_14	664.49	Graded suevite unit	140	29	-5.50	14.0	14.7
		68_1_67_70	692.13	Graded suevite unit	160	33	¤		
		90_2_48_50b	730.29	Granitoid clast inside UIM (Unit 3A)	780	59	-16.30	0.6	-15.0
		91_3_18_20	734.25	UIM (Unit 3A)	350	49	-2.30	2.1	1.8
		139_1_6_8	853	Dolerite	1100	61	-12.50	0.9	-10.3
		162_2_99.5_101.5	914.63	Dolerite	830	50	-1.60	0.5	-0.5
		192_1_56_58	997.65	LIMB	370	86	-20.10	2.9	-0.1
		273_2_78_80	1242	LIMB	180	29	-0.30	1.7	2.2
		276_3_93_95	1252.33	Metamorphic clast (gneiss/amphibolite)	170	22	0.70	1.4	2.5
		283_2_46_48	1272.97	LIMB	260	52	¤		
		303_3_17.5_19.5	1334.33	LIMB	360	25	¤		
Mexico	Yucatán-6	Y6_N14_P15	1208-1211	Suevite	52	1	¤		
	Yucatán-6	Y6_N17	1295.95-1299	Impact melt rock	24	0.1	¤		

[#] Sulfate specific isotope ratio was calculated using a simplified isotope dilution equation [$\delta^{34}S_{sulfate}$= $\delta^{34}S_{bulk}$-TRIS*($\delta^{34}S_{sulfide}$-$\delta^{34}S_{bulk}$)/(bulk S concentration-TRIS)] assuming that all the S that is not found in the reduced inorganic S phase in the samples is related to sulfates.

¤ Insufficient amount of material for reliable measurements.

Table S4. Bulk S, Co, Cr, and Ni concentrations; raw and background subtracted (BS) Ni/Cr ratios; and Ir and Re information values in different K-Pg boundary deposition sites. Error bars represent the standard deviation between two or more replicates.

Location	Section/Core	Sample ID	Depth cm	Lithological unit	Co µg g^{-1}	SD µg g^{-1}	Cr µg g^{-1}	SD µg g^{-1}	Ni µg g^{-1}	SD µg g^{-1}	Ni/Cr Raw	Ni/Cr BS	Ir ng g^{-1}	Re ng g^{-1}
Denmark	Stevns Klint A	SK KSA 50	27.5	K-Pg section	**4**	0	**38**	1	**42**	2	1.1	**1.1**		
	Stevns Klint A	SK KSA 40	19.5	K-Pg section	**6**	0	**74**	1	**58**	2	0.8	**0.7**		
	Stevns Klint A	SK KSA 43	16.5	K-Pg section	**26**	2	**50**	1	**88**	4	1.8	**1.8**		
	Stevns Klint A	SK KSA 45	14.5	K-Pg section	**22**	2	**56**	1	**95**	4	1.7	**1.7**		
	Stevns Klint A	SK KSA 47	12.5	K-Pg section	**34**	4	**74**	3	**325**	15	4.9	**5.0**		
	Stevns Klint A	SK KSA 20	9.5	K-Pg section	**21**	2	**85**	1	**310**	18	3.8	**3.8**		
	Stevns Klint A	SK KSA 23	7.5	K-Pg section	**32**	2	**92**	2	**464**	18	4.9	**5.0**		
	Stevns Klint A	SK KSA 2	0.75	K-Pg section	**33**	2	**202**	4	**457**	20	2.5	**2.5**		
	Stevns Klint A	SK KSA 1b	0.75	K-Pg section	**20**	1	**126**	1	**254**	11	2.1	**2.1**		
	Stevns Klint A	SK KSA 1	0.015	K-Pg section	**174**	13	**197**	3	**2288**	99	11.5	**11.6**		
	Stevns Klint A	SK KSB 98	-4.5	K-Pg section	**0**	0	**2**	0	**3**	0	1.3			
Spain	Caravaca	SM75 510	22	K-Pg section	**4**	0	**47**	2	**26**	2	0.6	**0.8**		
	Caravaca	SM75 507	7	K-Pg section	**28**	2	**159**	7	**184**	14	1.2	**1.4**		
	Caravaca	504	1	K-Pg section	**37**	3	**138**	6	**160**	12	1.1	**1.4**		
	Caravaca	503C	0.25	K-Pg section	**51**	4	**229**	10	**270**	21	1.2	**1.3**		
	Caravaca	503B	0	K-Pg section	**351**	26	**429**	19	**2065**	160	4.7	**5.1**		
	Caravaca	501A	-1	K-Pg section	**4**	0	**35**	4	**24**	3	0.7	**0.0**		
	Caravaca	SM75 515	-24	K-Pg section	**5**	0	**47**	1	**25**	1	0.6	**0.6**		
	Caravaca	SM75 517	-44	K-Pg section	**5**	4	**45**	0	**23**	2	0.5	**-0.2**		
Texas, USA	Brazos River	JV-BR 18	100	K-Pg section	**7**	1	**90**	5	**31**	2	0.3	**0.3**		
	Brazos River	JV-BR 41	75	K-Pg section	**11**	1	**110**	6	**44**	2	0.4	**0.4**		
	Brazos River	JV-BR 16	65	K-Pg section	**9**	1	**109**	6	**40**	2	0.4	**0.4**		
	Brazos River	JV-BR 14	50	K-Pg section	**9**	1	**102**	6	**37**	2	0.4	**0.3**		
	Brazos River	JV-BR 28	45	K-Pg section	**12**	0	**113**	6	**55**	3	0.5	**0.5**		
	Brazos River	JV-BR 9	40	K-Pg section	**8**	1	**95**	5	**34**	2	0.4	**0.3**		
	Brazos River	JV-BR 38	28	K-Pg section	**8**	1	**80**	5	**33**	2	0.4	**0.4**		
	Brazos River	JV-BR 49	18	K-Pg section	**10**	1	**98**	6	**36**	2	0.4	**0.3**		
	Brazos River	JV-BR 19	15	K-Pg section	**11**	1	**82**	2	**44**	2	0.5	**0.5**		
	Brazos River	JV-BR 68	13.4	K-Pg section	**0**	0	**85**	5	**50**	3	0.6	**0.7**		
	Brazos River	JV-BR 60	10.9	K-Pg section	**7**	1	**78**	4	**25**	1	0.3	**0.3**		
	Brazos River	JV-BR 30	5.5	K-Pg section	**8**	1	**66**	4	**30**	2	0.5	**0.5**		
	Brazos River	JV-BR 50	2.5	K-Pg section	**3**	0	**22**	1	**8**	0	0.4	**0.2**		
	Brazos River	JV-BR 51	1.5	K-Pg section	**3**	0	**25**	1	**9**	0	0.3	**0.2**		
	Brazos River	JV-BR 66	0	K-Pg section	**5**	0	**46**	3	**14**	1	0.3	**0.2**		
	Brazos River	JV-BR 36	-15	K-Pg section	**14**	2	**75**	12	**37**	11	0.5	**0.5**		
	Brazos River	JV-BR 29	-20	K-Pg section	**3**	0	**15**	1	**7**	0	0.5			
	Brazos River	JV-BR 35	-26	K-Pg section	**11**	1	**114**	7	**46**	2	0.4	**0.4**		
	Brazos River	JV-BR 22	-32	K-Pg section	**7**	1	**130**	7	**31**	2	0.2	**0.2**		
	Brazos River	JV-BR 33	-40	K-Pg section	**11**	1	**142**	5	**49**	3	0.4	**0.3**		
	Brazos River	JV-BR 20	-52.5	K-Pg section	**11**	1	**114**	7	**38**	2	0.3	**0.3**		
	Brazos River	JV-BR 6	-125	K-Pg section	**9**	1	**128**	3	**41**	2	0.3	**0.3**		
	Brazos River	JV-BR 7	-225	K-Pg section	**11**	1	**156**	9	**54**	3	0.4	**0.4**		
N-Dakota, USA	Tanis	X-2761_1_0-1cm	12.5	Paleocene siltstone	**3**	0	**23**	2	**14**	1	0.6		**3**	**1.0**
	Tanis	X-2761_2_1-2cm	11.5	Paleocene siltstone	**3**	0	**31**	4	**15**	1	0.5	**0.1**	**3**	**0.4**
	Tanis	X-2761_3_2-3cm	10.5	Paleocene siltstone	**5**	0	**37**	6	**26**	1	0.7	**0.9**	**1**	**0.7**
	Tanis	X-2761_4_3-4cm	9.5	Paleocene coal	**19**	0	**77**	2	**199**	1	2.6	**3.4**	**12**	**3.3**
	Tanis	X-2761_5_4-5cm	8.5	Paleocene coal	**22**	2	**65**	2	**146**	3	2.2	**3.1**	**6**	**0.6**
	Tanis	X-2761_6_5-6cm	7.5	Paleocene coal	**23**	1	**96**	6	**148**	7	1.5	**1.8**	**8**	**1.3**
	Tanis	X-2761_7_6-7.25cm	6.375	Paleocene coal	**18**	0	**70**	3	**91**	0	1.3	**1.6**	**22**	**6.0**
	Tanis	X-2761_8A_7.25-8cm	5.375	K-Pg tonstein	**22**	1	**25**	2	**20**	3	0.8	**2.7**	**5**	**0.5**
	Tanis	X-2761_8B_8-8.5cm	4.75	K-Pg tonstein	**4**	0	**19**	2	**12**	2	0.6	**0.6**	**2**	**0.0**
	Tanis	X-2761_8C_8.5-9cm	4.25	K-Pg tonstein	**3**	0	**25**	2	**16**	2	0.6	**0.7**	**4**	**0.4**
	Tanis	X-2761_9_9-10cm	3.5	Event deposit	**10**	0	**41**	4	**33**	1	0.8	**1.1**	**5**	**0.8**
	Tanis	X-2761_10_10-11cm	2.5	Event deposit	**10**	1	**36**	3	**34**	2	0.9	**1.5**	**4**	**0.3**
Haiti	Beloc	Goderis et al. (2013)[22]		K-Pg boundary clay	**15**	0	**51**	2	**55**	0	1.2			
Texas, USA	Brazos River	Goderis et al. (2013)[22]		K-Pg boundary clay	**9**	0	**69**	5	**34**	2	0.5			
Colorado, USA	Long Canyon, Raton Basin	Goderis et al. (2013)[22]		K-Pg boundary clay	**1**	0	**19**	1	**2**	0	0.1			
Wyoming, USA	Dogie Creek, Powder River Basin	Goderis et al. (2013)[22]		K-Pg boundary clay	**1**	0	**59**	6	**6**	1	0.1			
Montana, USA	Brownie Butte, Hell Creek area	Goderis et al. (2013)[22]		K-Pg boundary clay	**6**	0	**53**	4	**21**	1	0.4			
Montana, USA	Seven Blackfoot Creek, Hell Creek area	Goderis et al. (2013)[22]		K-Pg boundary clay	**7**	0	**21**	1	**19**	1	1.0			
Italy	Frontale, Umbria-Marche	Goderis et al. (2013)[22]		K-Pg boundary clay	**57**	1	**113**	8	**205**	10	1.9			
Italy	Fonte D'Olio, Umbria-Marche	Goderis et al. (2013)[22]		K-Pg boundary clay	**39**	1	**108**	6	**169**	1	1.7			
Tunisia	Siliana	Goderis et al. (2013)[22]		K-Pg boundary clay	**22**	0	**187**	13	**127**	6	0.7			
Tunisia	Elles	Goderis et al. (2013)[22]		K-Pg boundary clay			**93**	7	**154**	7	1.6			

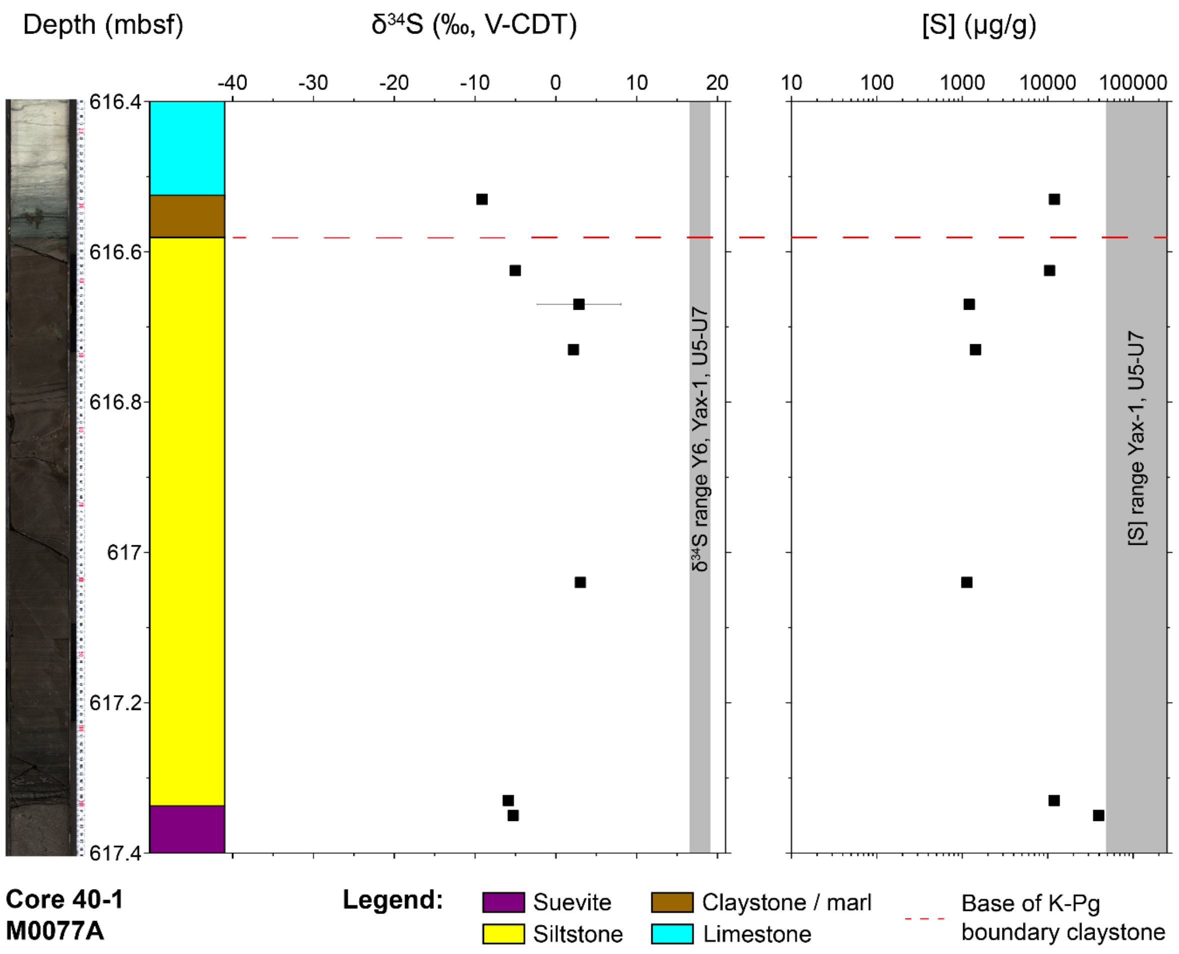

Fig. S1. S data within the K-Pg boundary claystone interval of the IODP-ICDP Expedition 364 M0077A drill core.

Detailed bulk $\delta^{34}S$ values and S concentrations of the K-Pg (Cretaceous-Paleogene) boundary claystone interval of the top part of the impactite sequence of the IODP-ICDP (International Ocean Discovery Program- International Continental Scientific Drilling Program) Expedition 364 M0077A drill core, focusing on drill core section 40R1 between 616.4 and 617.4 meters below sea floor (mbsf). Full profiles of the M0077A drill core can be found in Fig. 1B in the main manuscript.

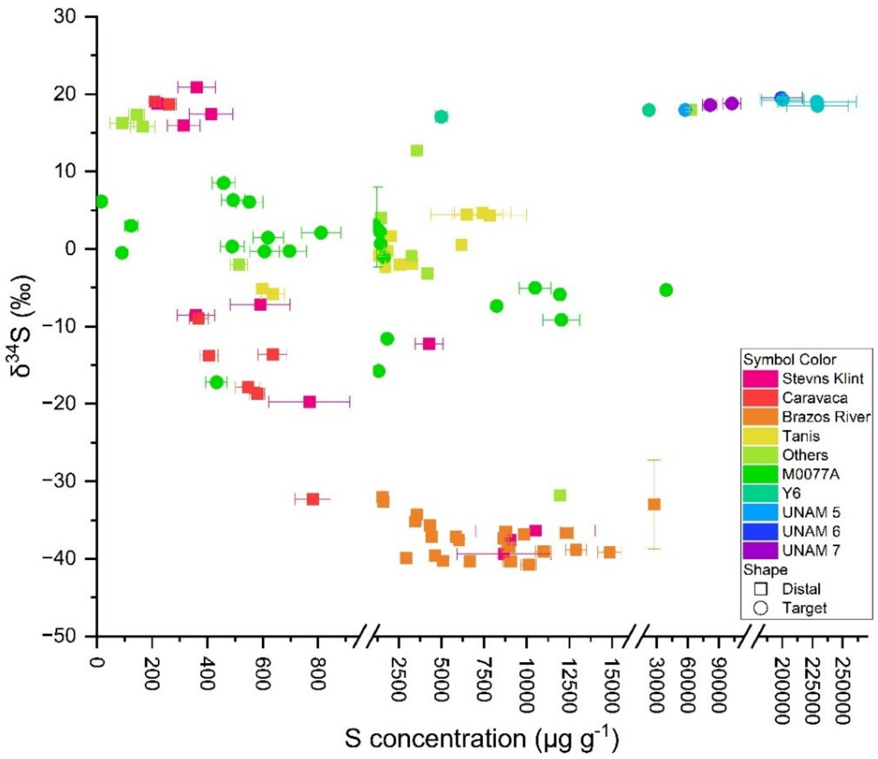

Fig. S2. S concentration vs. isotope ratio data for all drill cores and K-Pg boundary sites.

Bulk S concentration and S isotope ratio, expressed as $\delta^{34}S$, for all the impact target drill cores and K-Pg (Cretaceous-Paleogene) distal sites analyzed. It is suggested that the onshore impact target drill cores (Y6, Yucatán 6, from PEMEX, Petróleos Mexicanos; UNAM-5, UNAM-6, and UNAM-7 from UNAM, Universidad Nacional Autónoma de México; Yax-1, Yaxcopoil-1, from ICDP, International Continental Scientific Drilling Program) mostly have high S concentrations and $\delta^{34}S$ values, while the values for the offshore impact target drill core (M0077A from IODP-ICDP, International Ocean Discovery Program- International Continental Scientific Drilling Program, Expedition 364) cover a wider range of values. The former indicates high amounts of anhydrite, consistent with lithological observations, and the latter indicates inclusion of many different S species. For the M0077A drill core, values with medium S concentrations and low $\delta^{34}S$ values indicate influence of pyrite sedimentation, occurring during microbial reduction, while low S concentrations and $\delta^{34}S$ values near to zero indicate the influence of igneous rock. Similar observations can be made for the distal sites, with the inclusion of low S concentration and high $\delta^{34}S$ values, which indicate inclusion of marine sulfate and/or anhydrite deposition.

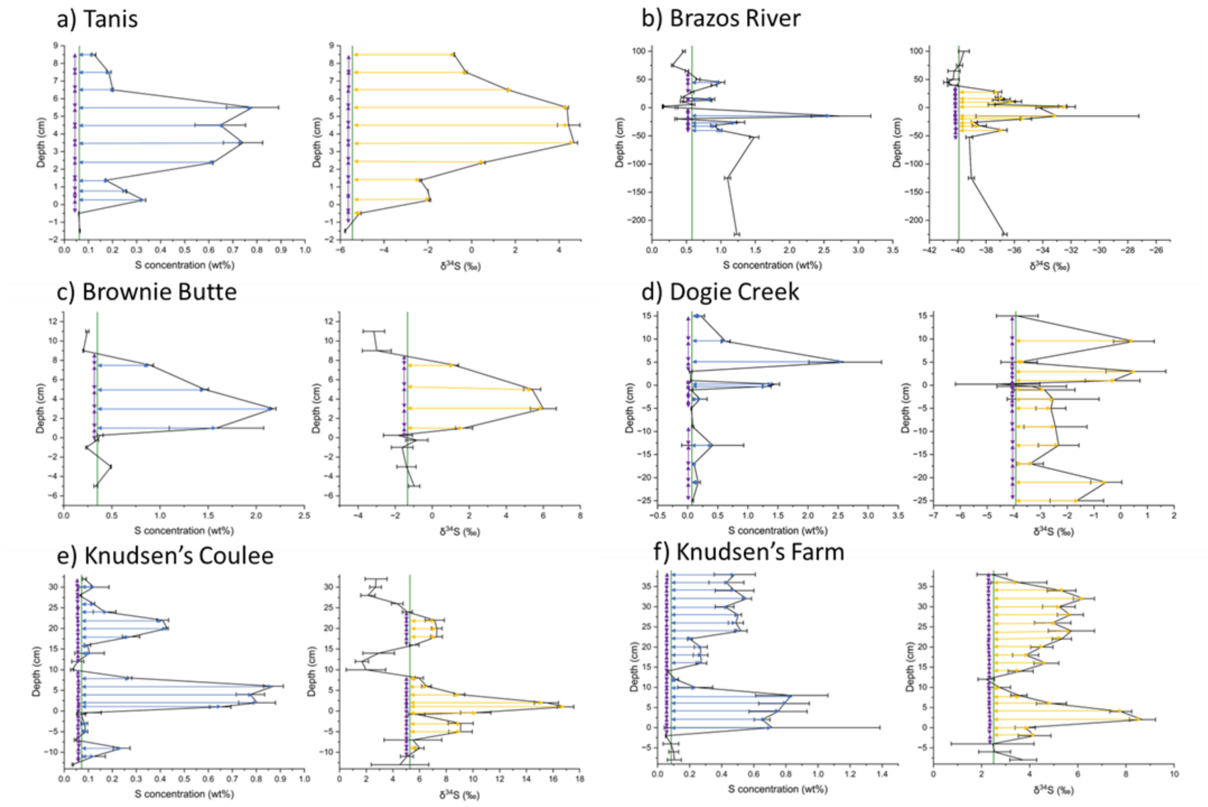

Fig. S3. S concentration and isotope ratio profiles for the different K-Pg boundary sites with background subtracted values marked out.

Bulk S concentration (blue) and S isotope ratio, expressed as $\delta^{34}S$, (yellow) profiles in the **a)** Tanis (based on values obtained in this study), **b)** Brazos River (based on values obtained in this study), **c)** Brownie Butte (based on values obtained from a previously published study[23]), **d)** Dogie Creek (based on values obtained from a previous published study[23]), **e)** Knudsen's Coulee Section (based on values obtained from a previous study[24]), and **f)** Knudsen's Farm Section (based on values obtained from a previous study[24]) K-Pg (Cretaceous-Paleogene) boundary impact event deposit. The error bars for the concentration correspond to 2SD and for $\delta^{34}S$ to expanded uncertainty. The green lines indicate the background values before the impact event deposition for the site, the purple lines indicate the vertical thickness of each sample unit ($VL_{Per\ sample}$), and the blue and orange lines indicate background-corrected S concentration ($C_{K-Pg\_site\_deposit}$) and $\delta^{34}S$ values ($\delta^{34}S_{K-Pg\_site\_deposit}$), respectively, as a result of the deposition. These data are used for the mass balance calculation to estimate the amount of impact-vaporized S.

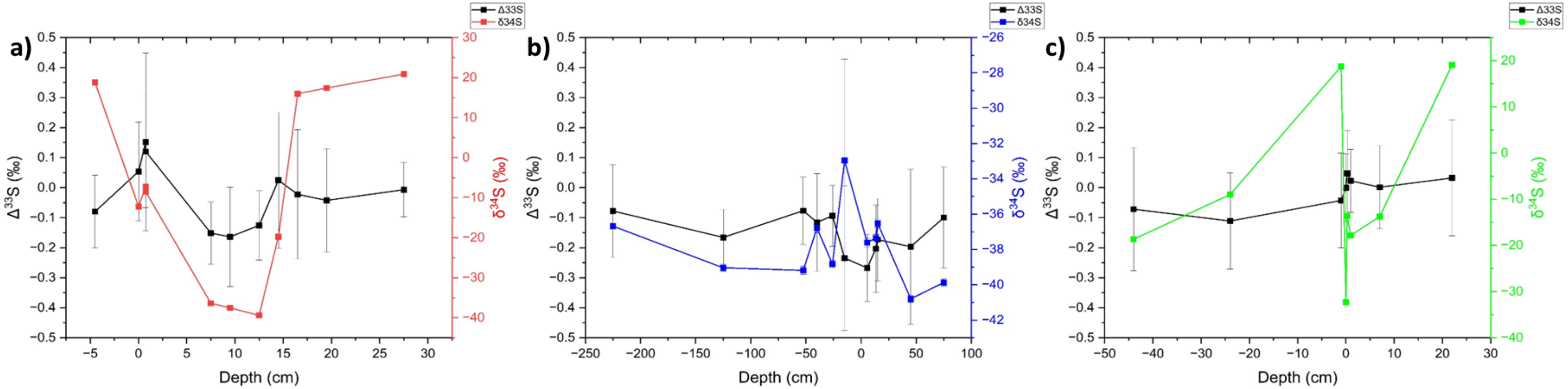

Fig. S4. $\delta^{34}S$ vs. $\Delta^{33}S$ for three K-Pg boundary sites.

Bulk $\delta^{34}S$ and $\Delta^{33}S$ (mass-independent fractionation tracer) in K-Pg boundary profiles at **a)** Stevns Klint (red), **b)** Brazos River (blue), and **c)** Caravaca (green) K-Pg (Cretaceous-Paleogene) boundary deposition sites. Error bars represent the external uncertainty and are sometimes smaller than the markers.

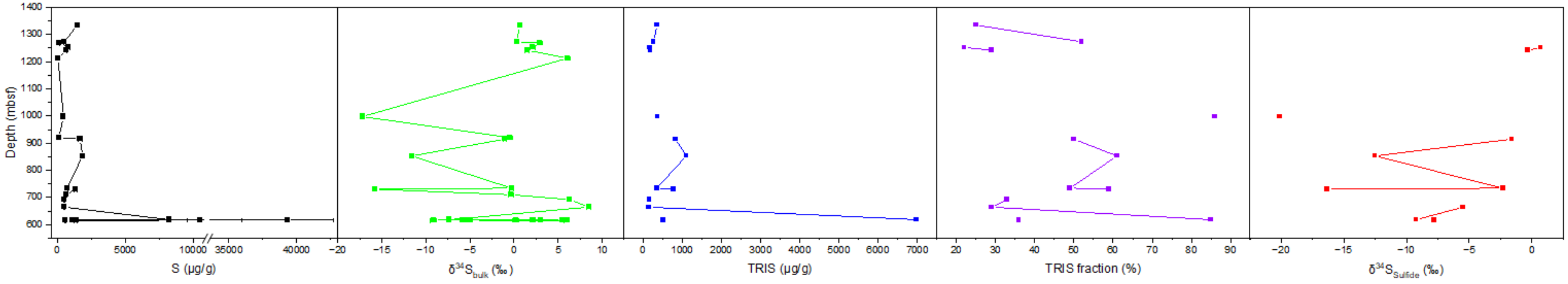

Fig. S5. Differences of S and TRIS concentration, TRIS-fraction, $\delta^{34}S$, and $\delta^{34}S_{sulfide}$ for the IODP-ICDP Expedition 364 M0077A drill core.

TRIS Bulk S concentration (black), bulk $\delta^{34}S$ (green), total reduced inorganic S (TRIS, blue), TRIS-fraction (TRIS/bulk S concentration*100, violet), and sulfide-specific isotope ratio ($\delta^{34}S_{sulfide}$, red) profiles of the IODP-ICDP (International Ocean Discovery Program- International Continental Scientific Drilling Program) Expedition 364 M0077A drill core.

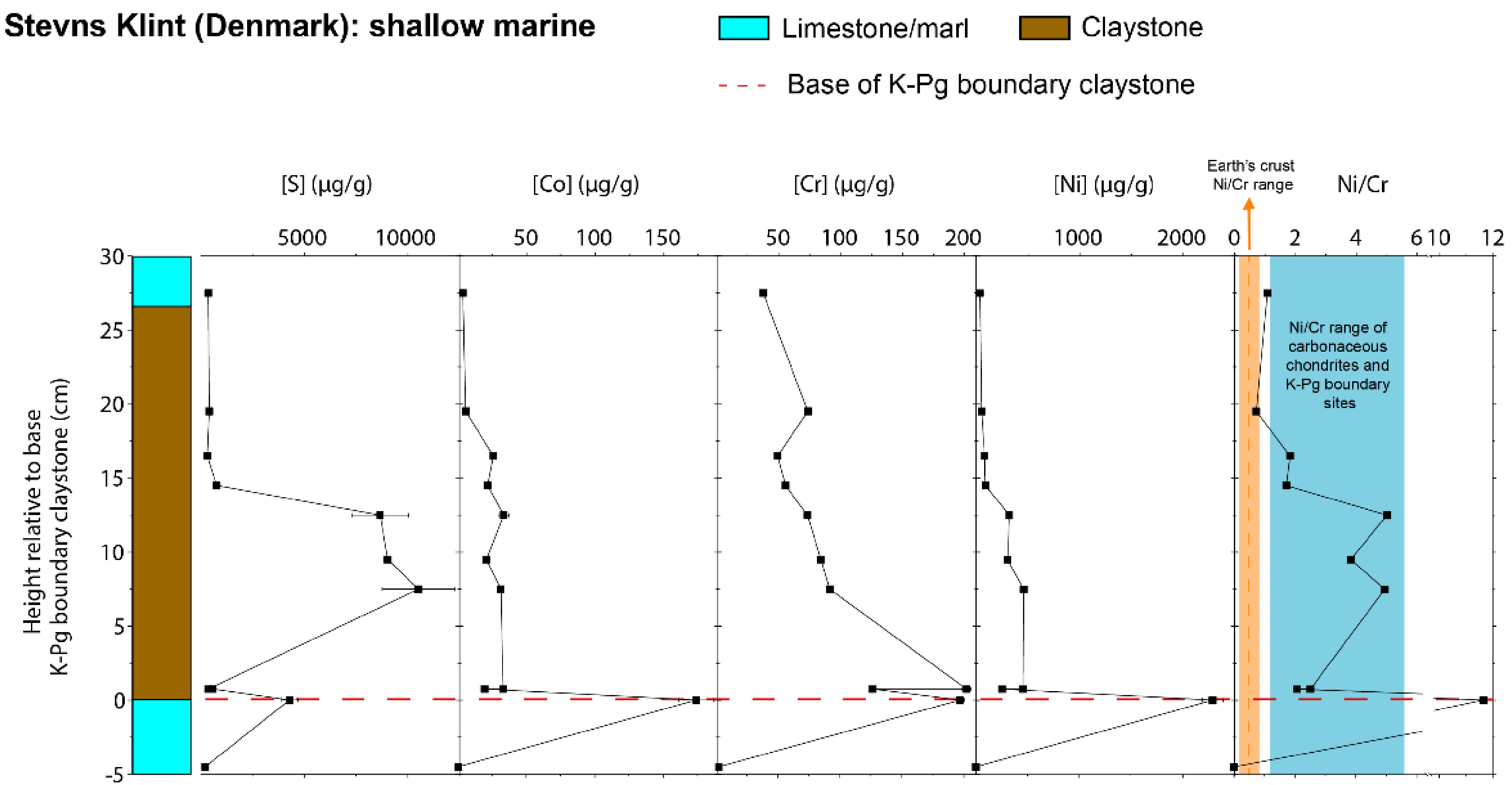

Fig. S6. Geochemistry of the Stevns Klint K-Pg boundary site.

Bulk S, Co, Cr, and Ni concentrations and background subtracted Ni/Cr ratios of the Stevns Klint K-Pg (Cretaceous-Paleogene) boundary site are presented. Error bars represent the standard deviation for two or more replicates and are often smaller than the markers. The dashed red line represents the base of the K-Pg boundary claystone, based on the 'start' of the sedimentological criteria, the presence of microkrystites, and the previously measured Ir anomaly[17]. Typical Ni/Cr values for the upper continental (UCC) crust[25] are marked with an orange dashed line and the range is marked by an orange interval. The blue region marks the previously published range for Ni/Cr values from other K-Pg boundary sites and for carbonaceous chondrites[22,26,27].

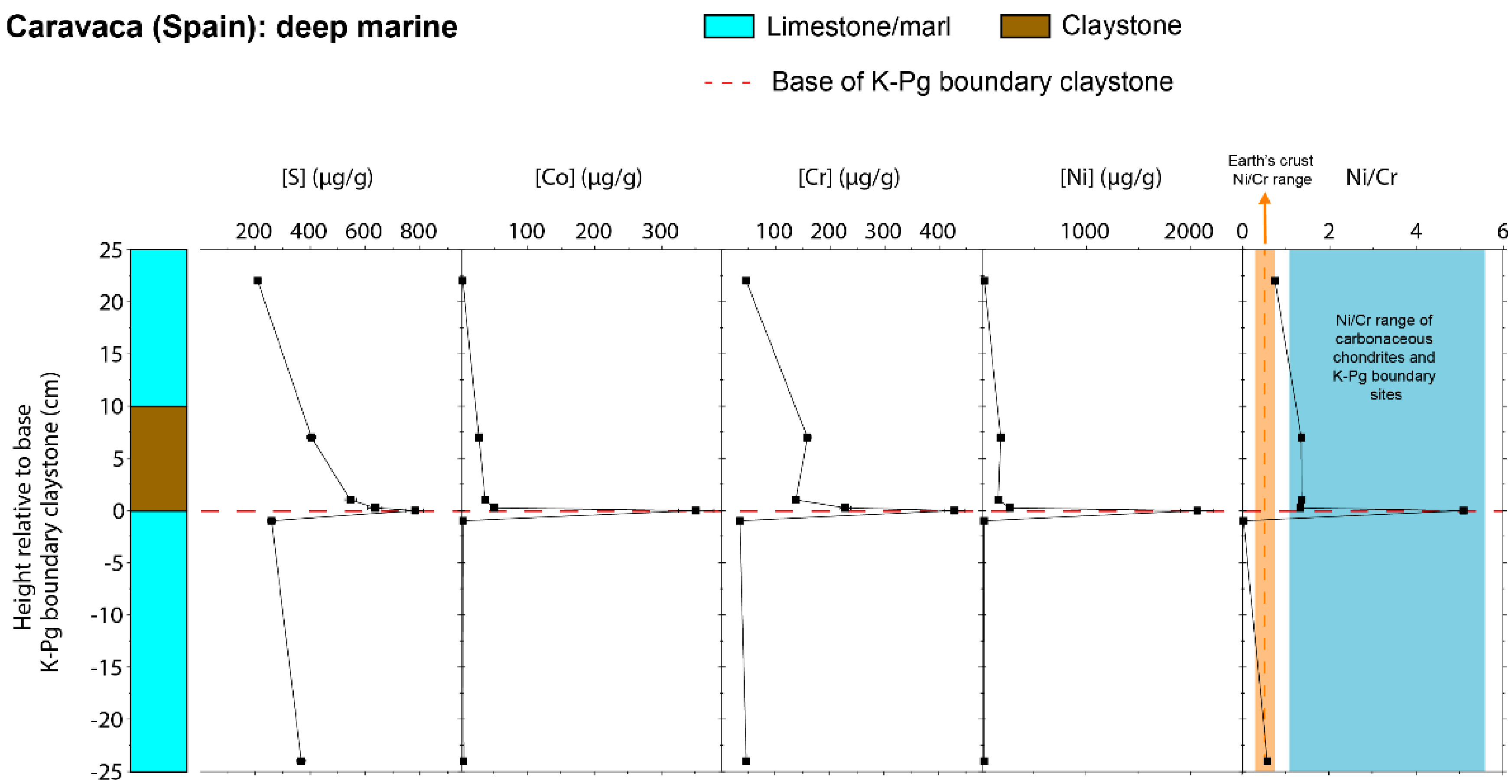

Fig. S7. Geochemistry of the Caravaca K-Pg boundary site.

Bulk S, Co, Cr, and Ni concentrations and background subtracted Ni/Cr ratios of the Caravaca K-Pg (Cretaceous-Paleogene) boundary site are presented. Error bars represent the standard deviation for two or more replicates and are often smaller than the markers. The dashed red line represents the base of the K-Pg boundary claystone, based on the 'start' of the sedimentological criteria, the presence of microkrystites, and the previously measured Ir anomaly[17]. Typical Ni/Cr values for the upper continental (UCC) crust[25] are marked with an orange dashed line and the range is marked by an orange interval. The blue region marks the previously published range for Ni/Cr values from other K-Pg boundary sites and for carbonaceous chondrites[22,26,27].

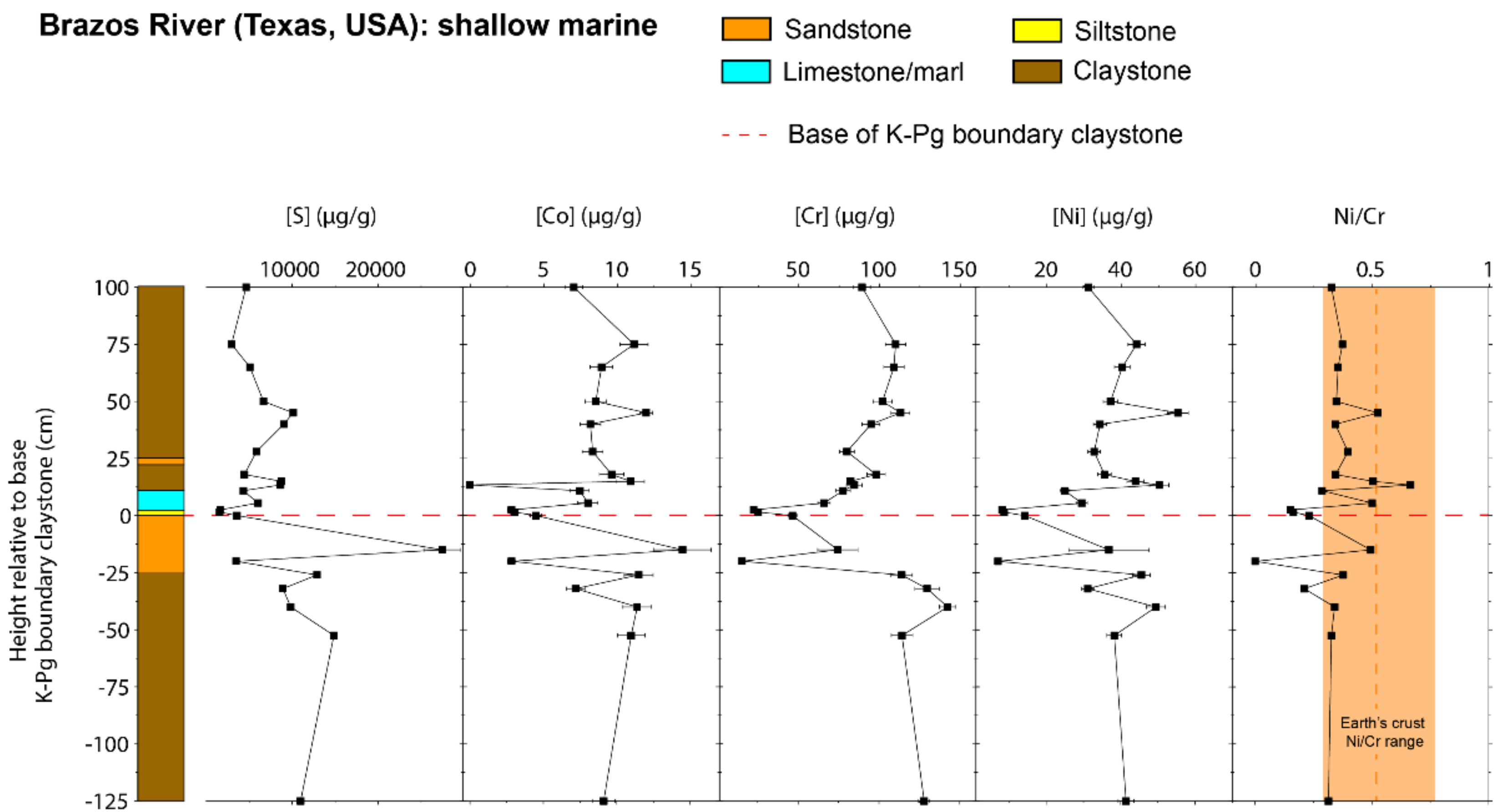

Fig. S8. Geochemistry of the Brazos River K-Pg boundary site.

Bulk S, Co, Cr, and Ni concentrations and background subtracted Ni/Cr ratios of the Brazos River K-Pg (Cretaceous-Paleogene) boundary site are presented. Error bars represent the standard deviation for two or more replicates and are often smaller than the markers. The dashed red line represents the base of the K-Pg boundary claystone, based on the 'start' of the sedimentological criteria and the previously measured Ir anomaly[17]. Typical Ni/Cr values for the upper continental (UCC) crust[25] are marked with a orange dashed line and the range is marked by an orange interval.

Supplementary References

1. Paytan, A. & Gray, E. T. Sulfur isotope stratigraphy. in *The Geologic Time Scale 2012* (eds. Gradstein, F. M., Ogg, J. G., Schmitz, M. & Ogg, G.) 167–180 (Elsevier B.V., 2012). doi:10.1016/B978-0-444-59425-9.00009-3.

2. Strauss, H. & Deutsch, A. The Chicxulub event—sulfur-bearing minerals and lithologies. *Geophysical Research Abstracts* **5**, (2003).

3. Paytan, A., Kastner, M., Campbell, D. & Thiemens, M. H. Seawater Sulfur Isotope Fluctuations in the Cretaceous. *Science* **304**, 1663–1665 (2004).

4. Gulick, S. P. S. *et al.* The first day of the Cenozoic. *Proc Natl Acad Sci U S A* **116**, 19342–19351 (2019).

5. Kaskes, P. *et al.* Formation of the crater suevite sequence from the Chicxulub peak ring: A petrographic, geochemical, and sedimentological characterization. *Bulletin of the Geological Society of America* **134**, 895–927 (2022).

6. Schaefer, B. *et al.* Microbial life in the nascent Chicxulub crater. *Geology* **48**, 328–332 (2020).

7. Kring, D. A., Whitehouse, M. J. & Schmieder, M. Microbial Sulfur Isotope Fractionation in the Chicxulub Hydrothermal System. *Astrobiology* **21**, 103–114 (2021).

8. Kring, D. A. *et al.* Probing the hydrothermal system of the Chicxulub impact crater. *Sci. Adv* **6**, 3053–3082 (2020).

9. Tino, C. J. *et al.* Are Large Sulfur Isotope Variations Biosignatures in an Ancient, Impact-Induced Hydrothermal Mars Analog? *Astrobiology* **23**, 1027–1044 (2023).

10. Wang, W. *et al.* Sulfur isotopic signature of Earth established by planetesimal volatile evaporation. *Nat Geosci* **14**, 806–811 (2021).

11. Labidi, J., Cartigny, P. & Moreira, M. Non-chondritic sulphur isotope composition of the terrestrial mantle. *Nature* **501**, 208–211 (2013).

12. Labidi, J., Cartigny, P., Hamelin, C., Moreira, M. & Dosso, L. Sulfur isotope budget (32S, 33S, 34S and 36S) in Pacific-Antarctic ridge basalts: A record of mantle source heterogeneity and hydrothermal sulfide assimilation. *Geochim Cosmochim Acta* **133**, 47–67 (2014).

13. Labidi, J. & Cartigny, P. Negligible sulfur isotope fractionation during partial melting: Evidence from Garrett transform fault basalts, implications for the late-veneer and the hadean matte. *Earth Planet Sci Lett* **451**, 196–207 (2016).

14. Kring, D. A. Hypervelocity collisions into continental crust composed of sediments and an underlying crystalline basement: Comparing the Ries (~24 km) and Chicxulub (~180 km) impact craters. *Chemie der Erde* **65**, 1–46 (2005).

15. Junium, C. K. *et al.* Massive perturbations to atmospheric sulfur in the aftermath of the Chicxulub impact. *PNAS* **119**, 1–7 (2022).

16. Goderis, S. *et al.* Globally distributed iridium layer preserved within the Chicxulub impact structure. *Sci Adv* **7**, 1–13 (2021).

17. Smit, J. The global stratigraphy of the Cretaceous-Tertiary boundary impact ejecta. *Annu. Rev. Earth Planet. Sci* **27**, 75–113 (1999).

18. Lin, M. *et al.* Five-S-isotope evidence of two distinct mass-independent sulfur isotope effects and implications for the modern and Archean atmospheres. *Proc Natl Acad Sci U S A* **115**, 8541–8546 (2018).

19. Endo, Y., Danielache, S. O. & Ueno, Y. Total Pressure Dependence of Sulfur Mass-Independent Fractionation by SO 2 Photolysis. *Geophys Res Lett* **46**, 483–491 (2019).

20. Glasspool, I. J. & Scott, A. C. Phanerozoic concentrations of atmospheric oxygen reconstructed from sedimentary charcoal. *Nat Geosci* **3**, 627–630 (2010).

21. Vellekoop, J. *et al.* Shelf hypoxia in response to global warming after the Cretaceous- Paleogene boundary impact. *Geology* **46**, 683–686 (2018).

22. Goderis, S. *et al.* Reevaluation of siderophile element abundances and ratios across the Cretaceous-Paleogene (K-Pg) boundary: Implications for the nature of the projectile. *Geochim Cosmochim Acta* **120**, 417–446 (2013).

23. Maruoka, T., Koeberl, C., Newton, J., Gilmour, I. & Bohor, B. F. Sulfur isotopic compositions across terrestrial Cretaceous-Tertiary boundary successions. *Geological Society of America* **Special Paper 356**, 337–344 (2002).

24. Cousineau, M. L. Tracing biogeochemical processes using sulfur stable isotopes: two novel applications. (University of Ottawa, Ottawa, Ontario, 2013).

25. Rudnick, R. L. & Gao, S. Composition of the Continental Crust. in *Treatise on Geochemistry* (eds. Holland, H. D. & Turekian, K. K.) 1–64 (Elsevier-Pergamon, Oxford, 2003). doi:10.1016/B0-08-043751-6/03016-4.

26. Ritter, X., Deutsch, A., Berndt, J. & Robin, E. Impact glass spherules in the Chicxulub K-Pg event bed at Beloc, Haiti: Alteration patterns. *Meteorit Planet Sci* **50**, 418–432 (2015).

27. Tagle, R. & Berlin, J. A database of chondrite analyses including platinum group elements, Ni, Co, Au, and Cr: Implications for the identification of chondritic projectiles. *Meteorit Planet Sci* **43**, 541–559 (2008).
